# Adsorptive Removal of Emerging Antibiotic Contaminants from Aquatic Environments Using Magnetically Modified Biochar

**DOI:** 10.3390/toxics14050400

**Published:** 2026-05-07

**Authors:** Habib Ullah, Durakshan Iqbal, Waqar-Un-Nisa, Jawaria Abid, Fiza Sarwar, Maria Ashfaq, Ahmed Mahmoud Ismail, Xin Pan, Boya Kuang

**Affiliations:** 1 School of Intelligent Electrical and New Energy, Hengxing University of Science and Technology, Qingdao 266042, China; habib901@zju.edu.cn (H.U.);; 2 Center for Interdisciplinary Research in Basic Science, International Islamic University Islamabad, Islamabad 44000, Pakistan; durakshaniqbal2020@gmail.com (D.I.);; 3 Isotope Application Division, Pakistan Institute of Nuclear Science and Technology (PINSTECH), Islamabad 45650, Pakistan; 4 Department of Earth and Environmental Sciences, Bahria University, H-11 Campus, Islamabad 44000, Pakistan; fizasarwar@bahria.edu.pk; 5 Pests and Plant Diseases Unit, College of Agricultural and Food Sciences, King Faisal University, Al-Ahsa 31982, Saudi Arabia; 6 School of Materials Science and Engineering, Beijing Institute of Technology, Beijing 100081, China

**Keywords:** levofloxacin, amoxicillin, antibiotics adsorption, isotherm and kinetic models, modified orange peel biochar, pharmaceutical waste

## Abstract

The widespread presence of pharmaceutical residues, particularly emerging antibiotics such as levofloxacin (LVX) and amoxicillin (AMOX), in aquatic environments poses serious risks to ecosystems and public health. In this study, magnetically modified biochar was synthesized from orange peel waste and evaluated for the percentage removal of LVX and AMOX from synthetic wastewater. The biochar was chemically modified with iron to enhance its adsorption capacity and facilitate magnetic separation. The physicochemical properties of raw and iron-modified biochar were characterized using Fourier-transform infrared spectroscopy (FTIR), scanning electron microscopy (SEM), and energy-dispersive X-ray spectroscopy (EDX). Batch adsorption experiments were conducted to investigate the effects of temperature, contact time, adsorbent dosage, pH, and initial antibiotic concentration on removal efficiency. Antibiotic concentrations were quantified using UV–Vis spectrophotometry. Batch adsorption experiments revealed that iron-modified biochar (FeMBC) significantly outperformed raw biochar (RBC) in antibiotic removal. Optimal removal efficiencies of 90% for AMOX and 92% for LVX were achieved at an adsorbent dosage of 0.1 g, antibiotic concentration of 10 mg L^−1^, contact time of 120 min, and temperature of 30 °C. Equilibrium data were best described by the Langmuir isotherm model, indicating monolayer adsorption, with correlation coefficients of 0.98 for AMOX and 0.97 for LVX. Kinetic analysis showed that the pseudo-second-order model provided the best fit, suggesting that chemisorption dominated the adsorption process. Thermodynamic studies confirmed that the adsorption was spontaneous and exothermic. Overall, the results demonstrate that iron-modified orange peel biochar is an efficient (90% better removal efficiency than RBC), low-cost, and environmentally sustainable adsorbent for the removal of emerging antibiotics from pharmaceutical wastewater, offering strong potential for practical water treatment applications.

## 1. Introduction

Organic pollutants pose a significant threat to the environment and public health due to their persistence, toxicological effects, and potential bioaccumulation in the ecosystem [1]. Pharmaceutical products, although indispensable for human and animal health, have emerged as environmental contaminants of emerging concern because of their widespread occurrence, chemical persistence, and incomplete removal by conventional wastewater treatment systems [2,3]. These compounds, which include antibiotics, analgesics, hormones, and anti-inflammatory agents [4], enter aquatic and terrestrial environments through municipal wastewater effluents, hospital discharges, pharmaceutical manufacturing effluents, and agricultural runoff [2,5]. Once released, many pharmaceuticals persist because they are only partially metabolized in humans and animals and exhibit low biodegradability under typical treatment conditions, thereby resisting removal during biological treatment processes [3]. The persistence of antibiotics in surface water, groundwater, and even drinking water sources is particularly concerning due to their potential to exert selective pressure on microbial communities, promoting the spread of antibiotic-resistance genes (ARGs) and antibiotic-resistant bacteria (ARB), which pose a direct threat to public health and ecosystem stability [2].

Several physicochemical and biological methods, including electrochemical processes, adsorption, filtration, chemical precipitation, ion exchange, reverse osmosis, and coagulation, are widely used for wastewater treatment; among these, adsorption is considered one of the most attractive approaches due to its eco-friendly nature, cost-effectiveness, operational simplicity, and high efficiency in removing diverse contaminants from polluted water [2,6,7]. Membranes are important in the pharmaceutical industry for the separation of antibiotics and salts [8]. Conventional treatment plants often fail to completely eliminate low-level pharmaceutical residues, which has driven increasing interest in adsorption-based technologies that perform effectively at low contaminant concentrations with relatively low energy requirements. In saline–alkali soils, poor aggregate stability limits water infiltration and root growth. Soil aggregates, as the soil matrix, are of paramount importance in enhancing soil quality and functionality [9]. Therefore, amendments that promote aggregation are highly desirable. Various locally available materials, including minerals, biological resources, and agricultural residues, have been explored as adsorbents, with carbon-based materials being particularly preferred [7]. Among these, biochar has gained significant attention as a sustainable and efficient adsorbent due to its porous structure, high surface area, and strong adsorption capacity. Biochar is produced through the carbonization of biomass feedstocks under oxygen-limited conditions, using materials such as sawdust, agricultural waste, coconut shells, woodchips, and wastewater sludge [10], and has demonstrated effectiveness in removing pollutants from both water and soil [11]. Common production methods include torrefaction, pyrolysis, microwave heating, gasification, and hydrothermal carbonization [12]. Its physicochemical properties—such as pore size distribution, surface area, cation exchange capacity, and surface functional groups—are key factors governing adsorption performance and vary depending on production conditions [13]. Compared to conventional activated carbon, biochar offers advantages such as sustainable production, tunable surface chemistry, and cost efficiency, while its ability to adsorb organic micropollutants, including antibiotics, is attributed to its diverse functional groups and porous architecture [3,14]. Moreover, biochar derived from agricultural waste supports circular economy principles by converting biomass residues into value-added adsorbents, thereby reducing environmental burdens and enhancing resource utilization [2,5].

Among modified forms, magnetically modified biochar (MBC) has emerged as a particularly versatile adsorbent because it couples enhanced adsorption capacity with magnetic separability, which simplifies recovery after treatment and supports reuse [15,16,17]. Magnetic biochar is typically fabricated by introducing iron species—such as Fe^2+^/Fe^3+^ salts or iron oxides—into the biochar matrix, improving surface chemistry and facilitating stronger interactions with contaminants [18]. Recent reviews of MBC for antibiotic removal report that numerous magnetic biochars achieve removal efficiencies exceeding 90% for a range of antibiotics (e.g., sulfamethoxazole, tetracycline, ciprofloxacin) within short contact times under optimal conditions [18]. These studies note that magnetic biochar not only enhances adsorption but can also act as a catalyst support, enabling coupling with advanced oxidation processes for simultaneous adsorption and degradation [19,20]. Research into iron-modified biochars, including those produced from diverse feedstocks such as waste biomass and agricultural residues, supports the conclusion that magnetic modification consistently improves adsorption performance relative to raw biochar [21].

Despite these advances, studies specifically comparing raw and iron-modified biochar derived from citrus wastes—such as orange peel—remain limited, especially under systematically optimized conditions for antibiotic removal. Therefore, the objectives of this study was to (i) synthesis of biochar from orange peel waste and modify it with iron to obtain magnetically modified biochar, (ii) compare the physiochemical characteristics of raw and modified biochar, (iii) evaluate and compare their adsorption performance for the removal of levofloxacin (LVX) and amoxicillin (AMOX) under varying operational conditions, and (iv) analyze the adsorption behavior using kinetic and isotherm models. This study aims to provide a more systematic understanding of the effect of iron modification on adsorption performance while highlighting the potential of citrus waste derived biochar for antibiotic removal.

## 2. Materials and Methods

### 2.1. Reagents and Material

The materials used in this study included oranges purchased from a local market. The oranges were thoroughly washed with distilled water available in the laboratory to remove any external contaminants, followed by peeling to obtain the orange peels. The peels were then air-dried for 48 h at room temperature to ensure complete moisture removal. The dried peels were subsequently ground into small pieces for the biochar preparation process.

For the iron modification of the orange peel-based biochar, iron salt (FeCl_3_) was obtained from Sigma-Aldrich (St. Louis, MO, USA) and used as the iron source. All the reagents used in this experiment were of analytical grade, and all solutions were prepared using distilled water to ensure purity and consistency in the experimental procedures.

### 2.2. Synthesis of Raw Biochar and Iron-Modified Biochar

Dried orange peels were subjected to pyrolysis at 350 °C for 30 min. The sample of raw biochar (RBC) was allowed to cool at room temperature in the furnace after pyrolysis. For the preparation of iron-modified biochar (FeMBC), 139 g of iron salt was mixed with 500 mL of distilled water to make 1 M solution. After that, the mixture was stirred for 15 min with a magnetic stirrer. 150 g of biochar was then added to the iron solution and followed by further stirring for 20 min. The mixture was allowed to heat in the oven for 24 h at 100 °C. The modified biochar sample was put in airtight bottles until it was used [7].

### 2.3. Characterization

The structure of RBC and FeMBC was investigated using Fourier-transform infrared spectroscopy (FTIR), Perkin Elmer, Springfield, IL, USA; Spectrum IR Version 10.6.2. The surface morphology of the samples was examined using scanning electron microscopy (SEM model KYKY-EM 6900). These samples were examined under various magnifications. X-ray diffraction (XRD model X Pert3 Panalytical Turkey) is used to reveal chemical composition information. Energy-dispersive X-ray (EDX) microanalysis is used to generate distinctive X-rays of the samples.

### 2.4. Adsorption Experiment

Levofloxacin (0.1 g) and 0.1 g of amoxicillin antibiotic were dissolved in 1 L of distilled water to prepare the stock solution 200 mg/L. Levofloxacin and amoxicillin were selected due to their widespread use, frequent detections in aquatic environments and persistence in wastewater effluents [22]. The adsorption studies of levofloxacin and amoxicillin with synthesized biochar composites were carried out in flasks by the batch method containing adsorbate solutions of known concentrations and a desired amount of RBC and FeMBC adsorbent at a speed of 120 rpm in a shaking incubator. Variable parameters such as contact time (40 min, 80 min, 120 min, 160 min and 200 min.), concentration of antibiotic (1 ppm, 5 ppm, 10 ppm, 15 ppm and 20 ppm), dosages of biochar (0.01, 0.05, 0.1, 0.5 and 1.5 g, 1.0 g and 1 g) and temperature (20 °C, 25 °C, 30 °C, 35 °C, and 40 °C) were used in the study. While studying a single parameter during the experiment, the rest of the parameters were kept constant. The concentrations of amoxicillin and levofloxacin were measured using a UV-Vis spectrophotometer (Shimadzu UV-1800, Kyoto, Japan) at the maximum absorbance wavelength of 228 nm and 280 nm, respectively [23,24]. All the experiments were repeated in triplicate, and the data was presented with mean ± SD.

For accurate measurement, calibration curves for levofloxacin and amoxicillin were constructed using standard solutions. The limit of detection (LOD) and limit of quantification (LOQ) were determined based on the standard deviation of the blank signal and the slope of the calibration curve. Sample preparation followed standard protocols to ensure consistency and reliability in all measurements.

The removal efficiency of antibiotics and the adsorption capacity of biochars were calculated by the following equations:

(1) $$\%\;\mathrm{Removal}\;=\;\frac{C_{O}-C_{f}}{C_{f}}\times 100$$

(2) $$\mathrm{Absorption}\;\mathrm{capacity}\;\mathrm{at}\;\mathrm{equilibrium},\;q_{e}=\frac{C_{O}-C_{e}}{m}V$$

where $C_{o}$ = initial concentration of antibiotic (ppm), $C_{f}$ = final concentration of antibiotic (ppm), $C_{e}$ = Equilibrium concentration of antibiotic (ppm), V = volume of antibiotic solution used (mL) and m = mass of adsorbent (g).

### 2.5. Adsorption Kinetic Model Studies

The adsorption process of the adsorbate onto the adsorbent was determined by the pseudo-first-order model and the pseudo-second-order by the following equations [25].

Pseudo-first-order: log (q_e_ − q_t_) = logq_e_ − k_1_t (3)

(4) $$\mathrm{Pseudo-second-order}:\;\frac{t}{q_{t}}=\frac{1}{k_{2}{qe}^{2}}+\frac{1}{q_{e}}t$$

where t = time, k_1_ = pseudo-first-order rate constant of adsorption (min^−1^), k_2_ = pseudo-second-order rate constant of adsorption (g/mg^.^ min), q_e_ = amount of antibiotic adsorbed at equilibrium (mg/g) and q_t_ = amount of antibiotic adsorbed at time.

### 2.6. Adsorption Isotherm Model Studies

The adsorption isotherms of antibiotics onto biochars were determined by the Freundlich and Langmuir isotherm models. The following equations are:

(5) $$\mathrm{Langmuir}\;\mathrm{isotherm}\;\mathrm{model}:\;\frac{C_{e}}{C_{q}}=\frac{C_{e}}{q_{m}}+\frac{1}{q_{mK_{L}}}$$

(6) $$\mathrm{Freundlich}\;\mathrm{isotherm}\;\mathrm{model}:\;{\mathrm{logq}}_{e}=\log\;K_{f}+\frac{1}{n}{\mathrm{logC}}_{e}$$

C_e_ = the equilibrium concentration after adsorption (mg/L), q_e_ = the amount of antibiotics adsorbed per gram, K_L_ = is the Langmuir constant (L/mg), q_m_ = maximum adsorption capacity of the isotherm model (mg/g), K_f_ = the Freundlich constant indicative of the relative adsorption capacity of biochar (mg/g) and 1/n = the adsorption intensity constant.

## 3. Results and Discussion

### 3.1. Biochar Characterization

#### 3.1.1. Fourier Transform Infrared (FTIR) Spectroscopy

**Figure 1:**
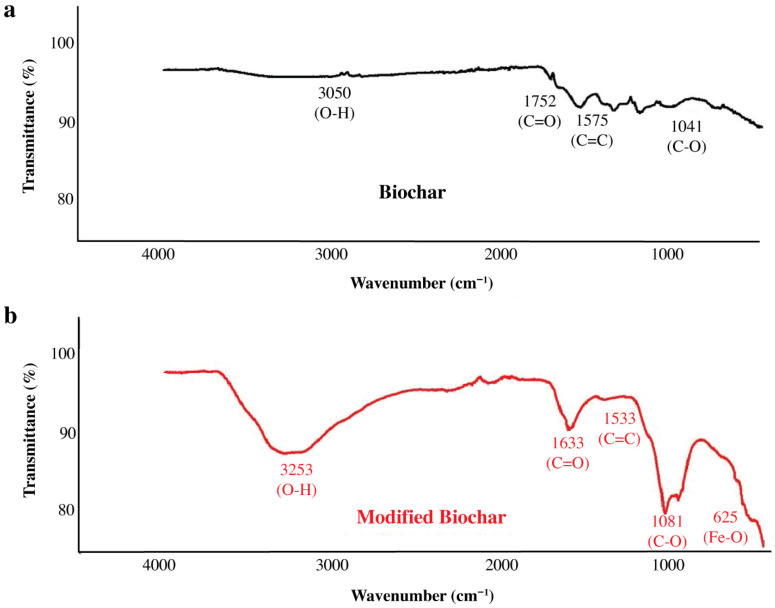
FTIR analysis of raw biochar (**a**), FeMBC (**b**).

The Fourier-transform infrared (FTIR) spectroscopy was used to evaluate the surface functional groups of raw biochar (RBC) and iron-modified biochar (FeMBC), as shown in Figure 1. The FTIR spectrum of RBC showed bands around 3060 cm^−1^ and 1752 cm^−1^, which may be associated with C-H stretching and carbonyl C=O stretching vibrations, respectively. These bands suggest the presence of oxygen-containing and aromatic functional groups on the biochar surface. After iron-modification, changes in the FTIR spectrum were observed, including bands in the regions around 1081 cm^−1^ and 625–633 cm^−1^; the peak near 1081 cm^−1^ may be attributed to C-O, C-O-C, or other oxygen-containing surface groups, while the lower wavenumber band around 625–633 cm^−1^ may be consistent with Fe-O related vibrations. However, FTIR bands in this region can overlap with other mineral or aromatic vibrations; this assignment should be considered tentative rather than definitive. Therefore, the FTIR results suggest changes in the surface chemistry of biochar after iron modification, but they do not alone provide complete confirmation of the exact iron bonding environment. Additional surface-sensitive analysis such as XPS, would be required to verify the chemical state of iron and its interaction with surface functional groups more conclusively. The observation of these distinctive peaks, especially the iron-related bands, is consistent with the findings reported by [26], who also demonstrated the successful modification of biochar with iron ions. The presence of these iron-associated peaks is further corroborated by the visual changes in the biochar’s surface morphology.

#### 3.1.2. Scanning Electron Microscopy (SEM)

**Figure 2:**
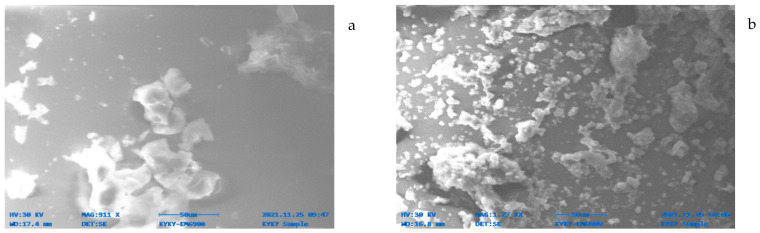
SEM analysis of RBC (**a**), FeMBC (**b**).

Scanning electron microscopy was used to observe the surface morphology of RBC (raw biochar) and FeMBC (iron-modified biochar), as shown in Figure 2a,b. The SEM image of RBC shows an irregular surface with rough edges and uneven particle morphology, which is consistent with the heterogenous structure of biomass-derived biochar. After iron modification, the FeMBC surface appears more fragmented and heterogeneous, with fine particulate deposits distributed across the biochar matrix. These observations indicate that the modification process altered the surface texture of the biochar and introduced additional morphological heterogeneity. The particulate features observed on the FeMBC surface may be associated with iron-containing species.

#### 3.1.3. Energy-Dispersive X-Ray

**Figure 3:**
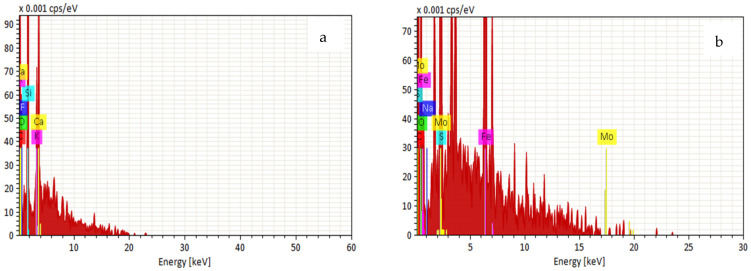
EDX analysis of RBC (**a**) and FeMBC (**b**).

Energy-dispersive X-ray spectroscopy was used to examine the elemental composition of RBC and FeMBC (Figure 3). The EDX spectrum of RBC showed that carbon and oxygen were the dominant elements as they are essential elements of cellulose materials, with approximate contents of 51 wt.% and 34 wt.%, respectively. This is consistent with the carbonaceous and oxygen-containing nature of biomass-derived biochar. Minor elements such as fluorine (1.41 wt.%), silicon (6 wt.%), potassium (3.17 wt.%), and calcium (3. 43 wt.%) were also detected, which may originate from the inherent mineral content of orange peel biomass or residual inorganic impurities. After iron modification, the EDX spectrum of FeMBC showed the presence of Fe (10.85 wt.%), indicating that iron-containing species were introduced on the biochar surface during the modification process. Carbon (32.12 wt.%) and oxygen (30.20 wt.%) remained major components of FeMBC, reflecting the underlying lignocellulosic biochar matrix [27]. Sodium (2.14 wt.%) and sulfur (2.94 wt.%) were also detected in smaller amounts and may be associated with residual chemicals or inorganic species introduced during preparation or washing. Molybdenum signal was also observed in the EDX spectrum; however, Mo was not used in any step of the synthesis or modification process and is not considered part of the FeMBC composition. So, this was excluded from further downstream analysis.

#### 3.1.4. X-Ray Diffraction (XRD) Analysis

**Figure 4:**
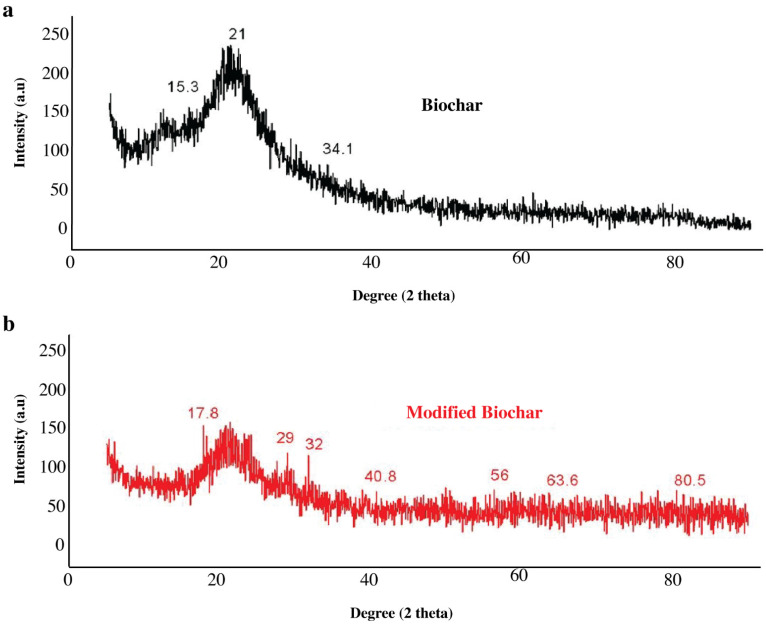
X-RD analysis of raw biochar (**a**) and FeMBC (**b**).

X-ray diffraction analysis was performed to examine the structural features of RBC and FeMBC (Figure 4). The XRD pattern of raw biochar displayed broad diffraction features, particularly around 21° and 34°, indicating the largely amorphous or poorly crystalline carbonaceous structure of the biochar. After iron modification, additional diffraction peaks appeared in the FeMBC pattern, including peaks near 29°, 32°, 40.8°, 56°, 63.6°, and 80.5° 2θ. These new peaks suggest the formation or deposition of crystalline iron-containing species on the biochar surface. However, assigning these peaks simply to “iron” would be insufficiently rigorous, because metallic iron and different iron oxide phases can show overlapping diffraction features. The observed peaks may be associated with iron oxide phases such as magnetite, maghemite, hematite, or related iron-containing crystalline species. Therefore, the XRD results support the presence of crystalline iron coning phases after modification, but the exact phase composition remains uncertain. Further analysis such as magnetic characterization would be required.

### 3.2. Adsorption Studies

The adsorption performance of raw biochar (RBC) was compared with iron-modified biochar (FeMBC) for the removal of amoxicillin (AMOX) and levofloxacin (LVX). All experimental parameters were systematically evaluated for both raw and modified biochar.

#### 3.2.1. Effect of Adsorbent Dosage

**Figure 5:**
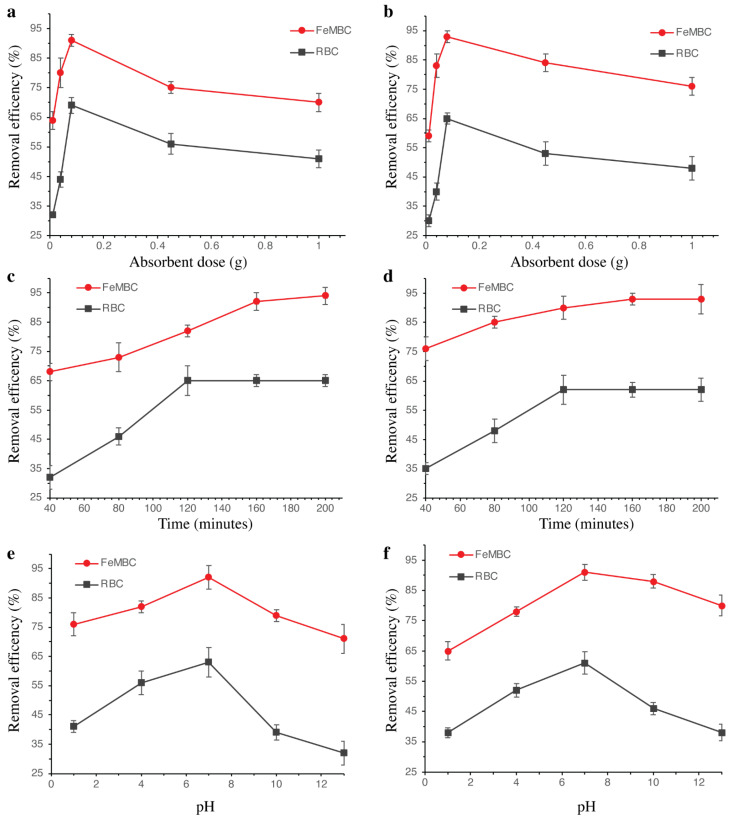
Effect of adsorbent dose, time, and pH on removal efficiency: (**a**) AMOX with RBC and FeMBC doses; (**b**) LVX with RBC and FeMBC doses; (**c**) contact time effect on AMOX removal; (**d**) contact time effect on LVX removal; (**e**) pH effect on AMOX removal; (**f**) pH effect on LVX removal. The experiment was repeated thrice, and the data was presented with mean ± SD.

Figure 5a,b illustrates the effect of adsorbent dose on the removal efficiency of AMOX and LVX using two different adsorbents, RBC and FeMBC. The removal efficiency of AMOX was evaluated for two adsorbents, RBC (represented by square markers) and FeMBC (represented by circular markers), across a range of adsorbent doses from 0.01 to 1 g. As shown in the graph, the removal efficiency of AMOX increases with the adsorbent dose for both adsorbents. At the lowest adsorbent dose (0.01 g), the removal efficiency was minimal, but as the dose increased, the efficiency of both adsorbents improved significantly. FeMBC demonstrated a sharp increase in removal efficiency, reaching 90% at a dose of 0.1 g, before experiencing a slight decrease as the dose approached 1 g. In contrast, RBC exhibited a more gradual increase in efficiency, with a peak removal efficiency of approximately 68% at the dose (0.1 g). This suggests that while both adsorbents effectively enhance AMOX removal, FeMBC is more efficient, particularly at lower doses. Similarly, the removal efficiency of LVX was assessed using the same adsorbents (RBC and FeMBC) across the same dose range. The removal efficiency for LVX exhibited trends similar to those observed for AMOX, with both adsorbents showing an increase in removal efficiency as the adsorbent dose was raised. FeMBC again displayed a rapid increase in efficiency, reaching nearly 92% at the lower doses of 0.1, with a slight decline at higher doses. The efficiency curve for RBC was less steep, gradually rising to around 65% at 0.1 g dose. A total of 0.1 g as the optimum adsorbent dose for both RBC and FeMBC was used as the fixed adsorbent amount in all subsequent experiments evaluating contact time and pH. These findings suggest that FeMBC is a more effective adsorbent for LVX as well, with higher removal efficiency achieved at lower doses compared to RBC. As in previous studies described that the amount of iron in biochar fabrication can regulate the adsorptive ability of FeBC obtained [28].

#### 3.2.2. Effect of Contact Time

Figure 5c,d illustrates the effect of contact time on the removal efficiency of AMOX and LVX using RBC and FeMBC adsorbents. The graph (c) shows the removal efficiency of AMOX over time, ranging from 40 to 200 min, for both RBC and FeMBC. As observed, the removal efficiency of AMOX increases with contact time for both adsorbents. For FeMBC, the efficiency rises sharply from 68% at 40 min to nearly 94% at 200 min. In contrast, RBC exhibits a slower increase in efficiency, reaching about 65% at 200 min. This suggests that RBC reached equilibrium earlier, while FeMBC showed higher adsorption capacity at longer contact times. Similarly, the removal efficiency of LVX was evaluated for both RBC and FeMBC adsorbents over the same time range. The removal efficiency of LVX follows a similar trend to that of AMOX but with some differences in the rate of increase. FeMBC shows a steep increase in efficiency, rising from around 76% at 40 min to nearly 93% at 200 min, indicating its rapid efficacy in removing LVX. On the other hand, RBC reaches a plateau in the efficiency curve after 120 min, stabilizing at about 60% removal, which is lower than that of FeMBC. The results suggest that FeMBC is more effective than RBC in removing LVX over the time period studied, with RBC requiring a longer time to reach its maximum removal efficiency. This trend is consistent with previous findings, where removal efficiency gradually increased with contact time, reaching approximately 90% after 160 min using sludge-based biochar [7].

#### 3.2.3. Effect of pH

Figure 5e,f shows the effect of pH on the removal efficiency of AMOX and LVX using RBC and FeMBC adsorbents. Graph (e) illustrates the removal efficiency of AMOX at varying pH levels (1, 4, 7, 10 and 13), for both RBC and FeMBC. The results indicate that the removal efficiency of AMOX is strongly influenced by the pH of the solution. For FeMBC, removal efficiency increases significantly as pH rises from 1 to 7, peaking at about 90% removal efficiency at pH 7. However, the efficiency drops drastically at higher pH levels, particularly after pH 8, suggesting that the adsorption capacity of FeMBC for AMOX decreases in highly alkaline conditions. In contrast, RBC shows a gradual increase in removal efficiency with increasing pH, reaching around 60% at pH 7, after which the efficiency decreases, with no significant improvement beyond pH 8. These findings indicate that FeMBC performs best in slightly acidic to neutral conditions compared to RBC. Similarly, graph (f) presents the removal efficiency of LVX across the same pH range for both RBC and FeMB adsorbents. Similar to the results for AMOX, FeMBC shows a sharp increase in removal efficiency, reaching nearly 91% at pH 7, after which the efficiency decreases as the pH continues to rise. For RBC, the removal efficiency increases gradually with pH, reaching about 62% at pH 7, then decreasing at higher pH values, similar to the trend seen for AMOX. This suggests that FeMBC is more efficient at slightly acidic to neutral pH, while RBC exhibits a relatively stable but less efficient performance across the tested pH range.

The effect of pH on adsorption may be better understood by considering the ionizable nature of the LVX and AMOX, which exist in different ionic forms depending on solution pH. Under acidic conditions, both antibiotics are expected to be predominantly protonated, whereas near neutral pH they may exist as zwitterions species, and under alkaline conditions they may undergo deprotonation. These pH-dependent changes could influence the electrostatic interactions between the adsorbent surface and the antibiotic molecules. The relatively higher adsorption observed around neutral pH suggests that favorable interactions may occur under these conditions, possibly due to reduced electrostatic repulsion and the contribution of non-electrostatic interactions, such as hydrogen bonding and π-π interactions. However, since these mechanisms were not directly confirmed experimentally, they should be considered as plausible interpretations rather than definitive adsorption pathways. Interestingly, a high pH of 12 (alkaline conditions) has been employed in AAMBRs for NaOH backwashing to regulate pH and mitigate membrane fouling, demonstrating that alkaline conditions can be beneficial in other wastewater treatment contexts [29].

#### 3.2.4. Effect of Concentration of Antibiotics

**Figure 6:**
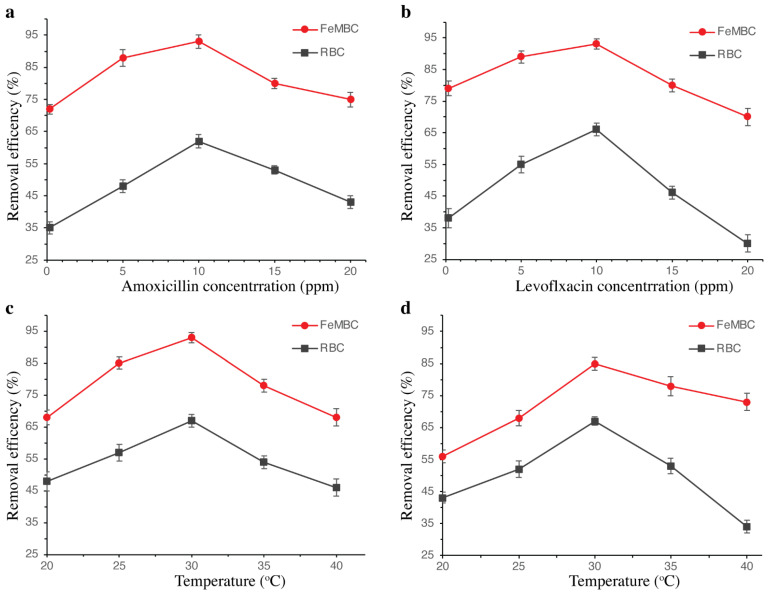
Effects of initial concentration of AMOX (**a**) and LVX (**b**), and temperature on AMOX (**c**) and LVX (**d**). The experiment was repeated thrice, and the data was presented with mean ± SD.

Figure 6a,b depicts the effect of varying antibiotic concentration on the removal efficiency of AMOX and LVX using RBC and FeMBC adsorbents. Graph (a) illustrates the removal efficiency of AMOX over a concentration range of 1, 5, 10, 15 and 20 ppm for both RBC and FeMBC. The results indicate that the removal efficiency initially increases with amoxicillin concentration, reaching a maximum efficiency of approximately 93% for FeMBC at 10 ppm and around 62% for RBC at the same concentration. Beyond 10 ppm, the removal efficiency decreases for both adsorbents. This trend suggests that at lower concentrations, the active sites of the adsorbents are sufficient for the adsorption process, leading to high removal efficiency. However, at higher concentrations, the saturation of adsorbent sites likely limits further removal, resulting in a decline in efficiency. FeMBC consistently exhibits higher removal efficiency than RBC across all concentrations, highlighting the enhanced adsorption capacity of iron-modified biochar.

Similarly, graph (b) in Figure 6 presents the removal efficiency of LVX over the same concentration range. Similar to AMOX, the removal efficiency for both RBC and FeMBC increases with LVX concentration up to 10 ppm, reaching peak efficiencies of approximately 92% for FeMBC and 67% for RBC. Beyond this concentration, the removal efficiency declines, indicating saturation of adsorption sites at higher antibiotic concentrations. The trend reinforces that FeMBC is a more effective adsorbent for LVX than RBC, capable of achieving higher removal efficiencies across the tested concentration range. The enhanced adsorption capacity of the modified biochar compared to the unmodified form can be attributed to the introduction of magnetic properties and additional active sites, which improve its affinity for antibiotic molecules [30,31].

#### 3.2.5. Effect of Temperature

The effect of temperature on the removal efficiency of AMOX and LVX by raw biochar and iron-modified biochar was investigated across a range of temperatures. As shown in Figure 6c, the adsorption of amoxicillin increased sharply at 30 °C, with removal efficiencies reaching approximately 76% and 92% for RBC and FeMBC, respectively. Similarly, maximum adsorption of levofloxacin was observed at 30 °C, with removal efficiencies of 68% and 92% for RBC and FeMBC, respectively (Figure 6d). These results are consistent with previous studies, where lower temperatures were found to enhance adsorption by expanding active sites, thereby increasing removal efficiency [32]. Conversely, at higher temperatures, adsorption decreased due to the limited availability of vacant active sites for the adsorbate [33].

### 3.3. Adsorption Isotherm Model Study

**Figure 7:**
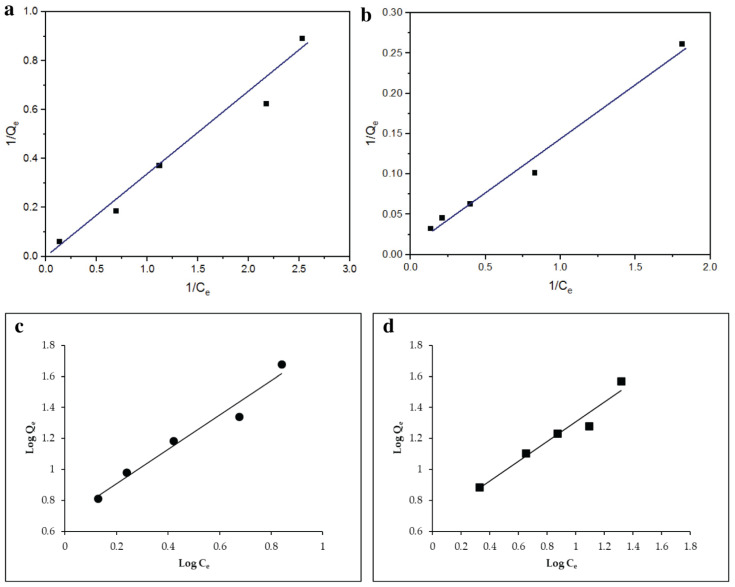
Langmuir isotherm model of AMOX (**a**) and LVX (**b**). Freundlich isotherm model of AMOX (**c**) and LVX (**d**).

The Langmuir adsorption isotherm, initially developed to describe gas–solid-phase adsorption on biochar, has long been a valuable tool for comparing and assessing adsorption performance. The results in Table 1 show that the Langmuir isotherm provided a slightly better fit to the experimental data than the Freundlich model, as indicated by the higher correlation coefficients for AMOX and LVX. This suggests that the adsorption behavior under the studied conditions is more consistent with the assumptions of the Langmuir model, which is commonly associated with adsorption on a finite number of relatively uniform sites. However, this model fit should not be interpreted as direct proof of purely monolayer adsorption, since isotherm models provide simplified representations of adsorption behavior and do not independently confirm the microscopic adsorption mechanism. The separation factor values, R_L_ < 1, further suggest that adsorption was favorable within the studied concentration range. In this context, normal adsorption is indicated by a Freundlich constant less than one. As pressure increases, the function approaches an asymptotic maximum. Additionally, a higher value of the Freundlich constant suggests greater heterogeneity in the adsorption process. In contrast, a value between 1 and 10 signifies that the isotherm model is desirable for characterizing the system. The correlation coefficients (R^2^) of 0.98 and 0.97 for AMOX and LVX (Table 1) highlight that antibiotic adsorption on FeMBC is favorable, with a strong fit to the model. These values indicate that the modified biochar has enhanced adsorption capabilities. Furthermore, under the same concentration, the adsorption of antibiotics by modified orange peel biochar increases with temperature. This suggests that the adsorption of AMOX and LVX on the modified biochar is facilitated by elevated temperatures (Figure 7).

The results in Table 1 demonstrate that the Langmuir isotherm provides the best fit for the data, supporting the monolayer adsorption of AMOX and LVX onto Fe MBC. The linear regression coefficients (R^2^ = 0.98 for AMOX and R^2^ = 0.97 for LVX) are higher than those of the Freundlich isotherm (R^2^ = 0.96 for AMOX and R^2^ = 0.95 for LVX), further confirming the suitability of the Langmuir model. Since the antibiotic removal rates (RL) are less than one, the adsorption process is more likely to occur.

### 3.4. Kinetic Adsorption for Antibiotics

**Figure 8:**
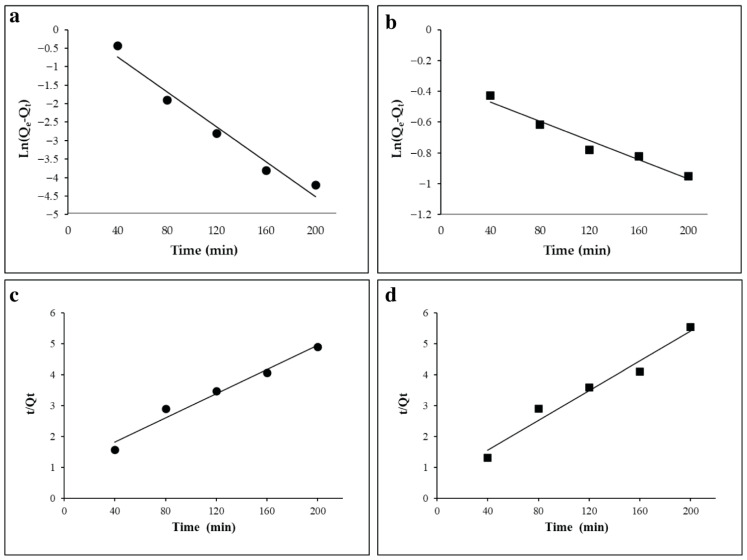
Pseudo-1st and -2nd-order models for the adsorption of AMOX (**a**,**c**) and LVX (**b**,**d**).

Figure 8 presents the adsorption kinetics of AMOX and LVX onto FeMBC, indicating a gradual increase in adsorption with contact time, reflecting a time dependent interaction between the adsorbate and adsorbent. The kinetic data were analyzed using pseudo-first-and pseudo-second order models, with the latter providing a better fit based on higher correlation coefficients (R^2^ = 0.98 and 0.96 for AMOX and LVX, respectively).

The pseudo-second-order model accounts for chemisorption processes, which involve the formation of strong chemical bonds between the adsorbent and the adsorbate. This model is known for its ability to predict the adsorption behavior in systems where the rate-limiting step is the chemical interaction. These findings suggest that the pseudo-second-order model better describes the adsorption kinetics and is better explained by a chemical process. In contrast, when applying the pseudo-first order kinetic model, which assumes that the rate of adsorption is proportional to the number of available adsorption sites, the fit was less accurate. The R^2^ values for the pseudo-first-order model were 0.95 and 0.94 for AMOX and LVX, respectively, which, although still relatively high, were lower than those obtained for the pseudo-second-order model. This suggests that the adsorption of amoxicillin on FeMBC is better explained by a chemical process, rather than a physical one, as the pseudo-second-order model more accurately represents the observed adsorption behavior. Although kinetic models provide a good fit, they do not account for mass transfer effects such as film or intraparticle diffusion, which may also influence adsorption rates. Therefore, the mechanism cannot be attributed solely to surface reactions, and further diffusion-based analysis is needed for a complete understanding.

### 3.5. Thermodynamic Isotherm Model Study

The thermodynamic characteristics of an adsorption process were evaluated to examine the temperature dependence and feasibility of AMOX and LVX adsorption onto Fe-modified orange peel biochar. Thermodynamic factors such as enthalpy (ΔH°), entropy (ΔS°), and Gibbs free energy (ΔG°) are essential and provide useful information on whether the adsorption process is thermodynamically favorable and whether heat is absorbed or released during adsorption.

**Figure 9:**
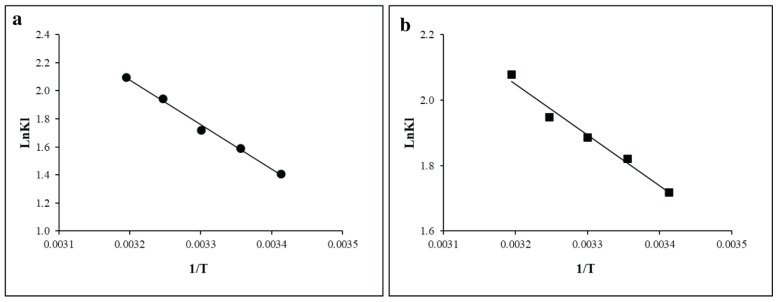
Thermodynamic model for the adsorption of AMOX (**a**) and LVX (**b**).

The negative values of ΔG° obtained for both AMOX and LVX indicate that the adsorption process was thermodynamically favorable under the temperature range. However, these values should not be interpreted as direct evidence of a specific adsorption mechanism. For AMOX, the ΔG° values remained negative across all tested temperatures, although the degree of favorability did not increase consistently with increasing temperature. This suggests that AMOX adsorption was spontaneous under the studied conditions, but its temperature dependence may be influenced by multiple interacting factors. For LVX the increasingly negative ΔG° values with increasing temperature suggest that adsorption became more thermodynamically favorable at higher temperatures. The calculated ΔH° values provide insight into the heat effect associated with adsorption. Based on the presented thermodynamic parameters, the negative ΔH° values suggest an exothermic nature of the adsorption process. Nevertheless, this interpretation should be considered together with the experimental adsorption trends and the Van’t Hoff fitting, since inconsistencies between adsorption capacity, ΔG° variation, and ΔH° sign may indicate limitations in model assumptions or calculation sensitivity. Additionally, the positive ΔS° values suggest increased randomness at the solid–solution interface during the adsorption process, which may be associated with structural rearrangement of water molecules and adsorbate species near the biochar surface. This suggests that the adsorption of AMOX and LVX on the modified biochar enhances the unpredictability at the interface, reflecting the complexity of the interactions between the adsorbate and the adsorbent (Figure 9).

While this study presents promising results, there are certain limitations that need to be addressed in future research. One of the key limitations is that the experiments were conducted using synthetic wastewater, which may not accurately reflect the complexity of real-world wastewater compositions. Specifically, the influence of competing ions, organic matter, and other contaminants commonly found in actual effluents was not evaluated. These factors could potentially affect the adsorption performance and may lead to different results when applied to real wastewater treatment systems. Moreover, the long-term stability and regeneration capacity of the adsorbent material used in this study were not thoroughly examined. The ability of the biochar to maintain its adsorption efficiency over extended periods, as well as its potential for regeneration and reuse, are critical factors for assessing its practicality in large-scale applications. To address these gaps, future research should focus on pilot-scale studies to evaluate the feasibility and efficiency of the adsorption process in real-world conditions. This includes the treatment of actual pharmaceutical wastewater, where the presence of diverse contaminants may impact the performance of the adsorbent. Additionally, long-term studies are essential to assess the durability, regeneration potential, and overall sustainability of the adsorbent material over multiple adsorption cycles.

## 4. Conclusions

In this study, biochar derived from orange peel waste was synthesized, modified with iron, and evaluated for the removal of levofloxacin (LVX) and amoxicillin (AMOX) from aqueous solutions. The available characterization results indicated changes in surface morphology, functional groups, and elemental composition after modification, while adsorption experiments showed that FeMBC achieved a higher removal efficiency (90%) than raw biochar under the tested conditions. These findings suggest that iron modification indirectly improved the adsorption performance of biochar.

The adsorption behavior was influenced by operational parameters such as temperature, contact time, adsorbent dosage, and initial antibiotic concentration. Although kinetic and isotherm models provided a good fit to the experimental data, underlying adsorption mechanisms, the contribution of mass transfer processes, and the specific role of iron-related surface interactions were not fully confirmed. This limitation is mainly due to the absence of advanced physiochemical characterization techniques such as BET surface area analysis, XPS surface chemical analysis, and magnetic characterization. Therefore, the proposed explanation for the enhanced performance of FeMBC should be regarded as a plausible interpretation rather than a fully established mechanism. Overall, the findings of this study indicate that biochar synthesized from orange peel, particularly after iron modification, has potential as a low-cost and environmentally friendly adsorbent for the removal of emerging antibiotic contaminants from wastewater. The use of agricultural waste as a precursor also supports the sustainability of this approach. However, further studies incorporating advanced surface area, surface chemistry, magnetic property, regeneration and long-term performance analysis are required before definitive conclusions can be made regarding the adsorption mechanism, structure activity relationships, and practical applicability of magnetically modified biochar in pharmaceutical wastewater treatment. These results provide a foundation for future research on large-scale implementation and long-term performance.

## Figures and Tables

**Table 1:** Isothermic, thermodynamic and kinetic parameters for the adsorption of AMOX and LVX onto iron-modified biochar.

a. Isothermic Parameters								
Langmuir	AMOX		LVX		Freundlich		AMOX	LVX
q_max_ (mg/g)	103.09		81.3		K_f_		4.78	4.67
K_L_ (L/mg)	0.072		0.038		1/n		1.1	0.65
RL	0.58		0.23		R^2^		0.96	0.95
R^2^	0.98		0.97					
**b. Thermodynamic Parameters**								
	**∆G° (k Jmol^−1^)**		**∆H° (k Jmol^−1^)**		**∆S° (k Jmol^−1^)**		**R^2^**	
**Temp (°C)**	**AMOX**	**LVX**	**AMOX**	**LVX**	**AMOX**	**LVX**	**AMOX**	**LVX**
20	−4.7349	−4.46395	−26.3969	−12.911	101.68	58.364	0.99	0.97
25	−5.2108	−4.6356						
30	−4.3859	−4.82918						
35	−3.9743	−4.986131						
40	−3.3468	−5.40764						
**c. Kinetics Parameters**								
**Order of Reaction**	**Parameters**	**AMOX**	**LVX**	**Order of Reaction**		**Parameters**	**AMOX**	**LVX**
	q_e_, exp (mg/g)	51.8	41.3					
Pseudo-first-order	q_e_, cal (mg/g)	1.40	1.13	Pseudo-second-order		q_e_, cal (mg/g)	50	41.66
	k1 (min^−1^)	0.000015	0.0001			k2 (g/mg.min)	0.01	0.0093
	R^2^	0.95	0.94			R^2^	0.98	0.96

## Data Availability

The data that support the findings of this study are available from the corresponding author upon reasonable request.
